# Can Carob-Fruit-Extract-Enriched Meat Improve the Lipoprotein Profile, VLDL-Oxidation, and LDL Receptor Levels Induced by an Atherogenic Diet in STZ-NAD-Diabetic Rats?

**DOI:** 10.3390/nu11020332

**Published:** 2019-02-03

**Authors:** Adrián Macho-González, Alba Garcimartín, María Elvira López-Oliva, Baltasar Ruiz-Roso, Isabel Martín de la Torre, Sara Bastida, Juana Benedí, Francisco José Sánchez-Muniz

**Affiliations:** 1 Nutrition and Food Science Department (Nutrition), Pharmacy School, Complutense University of Madrid, Plaza Ramón y Cajal s/n, 28040 Madrid, Spain; amacho@ucm.es (A.M.-G.); ruizrojo@ucm.es (B.R.-R.); ismart02@ucm.es (I.M.d.l.T.); sbastida@ucm.es (S.B.); 2 Pharmacology, Pharmacognosy and Botany Department, Pharmacy School, Complutense University of Madrid, Plaza Ramón y Cajal s/n, 28040 Madrid, Spain; a.garcimartin@ucm.es (A.G.); jbenedi@ucm.es (J.B.); 3 Departmental Section of Physiology, Pharmacy School, Complutense University of Madrid, Plaza Ramón y Cajal s/n, 28040 Madrid, Spain; elopez@ucm.es

**Keywords:** STZ-NAD, diabetes, carob fruit, functional meat, lipoproteins, LDL-receptor, lipoprotein oxidation

## Abstract

Carob fruit extract (CFE) has shown remarkable in vitro antioxidant properties and reduces postprandial hyperglycemia and hyperlipidemia in healthy animals. Development of functional meat products that contain bioactive components are presented as a great nutritional strategy. Until now, the effect of the consumption of restructured meat enriched with CFE in a murine model of diabetes has not been investigated. The objective of this study was to evaluate the effect on glycemia, lipemia, lipoprotein profile, *Ldlr*, arylesterase (AE), and very low-density lipoproteins (VLDL) and liver oxidation in streptozotocin-nicotinamide (STZ-NAD) growing Wistar diabetic rats fed restructured meat in the frame of a high cholesterol/high saturated-fat diet. In the present study, three groups (D, ED and DE) were fed cholesterol-enriched (1.4% cholesterol and 0.2% cholic acid) and high saturated-fat diets (50% of total energy from fats and 20.4% from saturated fatty acids). Rats were subjected to a STZ-NAD administration at the 3rd week. Group D did not receive CFE, while ED and DE rat groups received CFE before and after the diabetic induction, respectively. After eight weeks, D rats showed hyperglycemia and hypercholesterolemia, an increased amount cholesterol-enriched VLDL (β-VLDL), IDL and LDL particles and triglyceride-enriched HDL. ED and DE partially blocked the hypercholesterolemic induction with respect to D group (*p* < 0.001) and improved glycemia, cholesterol levels, lipoprotein profile, *Ldlr*, plasma AE activity and liver oxidation (*p* < 0.001). Fecal fat, moisture and excretion were higher while dietary digestibility was lower in ED and DE vs. D counterparts (*p* < 0.0014). In conclusion, CFE-enriched meat shows, for the first time, hypoglycemic and hypolipidemic effects in STZ-NAD animals fed high cholesterol/high saturated-fat diets. Likewise, it manages to reverse possible diabetes lipoprotein alterations if CFE-enriched meat is consumed before pathology development or improves said modifications if Type 2 Diabetes Mellitus is already established.

## 1. Introduction

Meat products are widely consumed in developed countries because they are a great source of protein, vitamins, and minerals [1]. However, it has been observed that a high intake of processed meat products or red meat greatly increases type 2 diabetes mellitus (T2DM) risk [2,3]. Although T2DM patients partially lose their ability to secrete insulin in response to carbohydrates due to insulin-resistance, they improve this hormone secretion when a meal contains high protein and amino acid levels [4,5].

On the other hand, T2DM patients usually suffer from dyslipemia, which includes quantitative, qualitative and kinetic lipoprotein abnormalities that, together, result in a shift towards a more atherogenic lipid profile [6,7]. These alterations are generally characterized in man by an increase in the level of very low-density lipoproteins (VLDL), small and dense low-density lipoproteins (LDL), as well as a high content of triglycerides in LDL and high-density lipoproteins (HDL), and an increased susceptibility of LDL to oxidation [6,8]. In previous studies, we observed that dietary cholesterol greatly increased the amount of plasma cholesterol in rodents giving rise to an increase of VLDL and LDL-cholesterol-enriched particles and to a decrease of HDL-cholesterol [9,10,11,12,13]. However, the effect of a high-cholesterol/high fat/high saturated fat diet in a T2DM model has been scarcely tested.

A key factor for the treatment of this pathology is dietetic intervention, and numerous studies have linked functional food consumption with an improvement of T2DM [14,15]. Thus, some functional foods, through the bioactive compounds they contain, could affect carbohydrate metabolism, giving rise to less marked postprandial hyperglycemia, improved pancreatic β-cell function and insulin secretion, as well as decreased insulin resistance [16]. Moreover, they could regulate lipid and lipoprotein metabolism and adipose tissue metabolism, modulate oxidative/antioxidative balance and inflammatory processes, improve weight management and prevent micro- and macro-vascular complications [17,18,19].

Our group has been working for years in functional foods and has extensive experience in the qualitative/quantitative modification of meat matrices with different active compounds, aimed at decreasing the potential negative effects on cardiovascular disease (CVD) risk factors of a high meat product consumption [9,10,11,12,13]. Thus, we have confirmed that the consumption of restructured meat enriched with walnut paste improves the antioxidant and lipoprotein profiles of at-risk CVD volunteers [20]. Similar data were also observed in Wistar rats fed restructured pork with *Himanthalia elongata* added [10]. Likewise, restructured pork enriched with silicon was able to partially block dyslipidemia induced by an obesogenic diet and reduce VLDL oxidation and improve liver arylesterase (AE) activity [9]. Now we have focused our attention on the development of a meat product enriched with carob fruit extract (CFE) because we have proven that it has hypoglycemic and hypolipemic properties in healthy animals [21,22]. Likewise, although CFE has been found to reduce oxidative alterations during meat storage [23], and to work as an antioxidant at frying temperatures [24], as far as we know, CFE has never been tested as a potential functional ingredient for meat products addressed at improving lipoprotein composition in streptozotocin plus nicotinamide (STZ-NAD) rats as an animal model of diabetes [25,26].

CFE is a purified fraction of carob pulp of the *Ceratonia siliqua L*. variety. Its composition is mainly based on 71–81% insoluble fiber [27]. CFE has a large amount of insoluble polyphenols and specifically high molecular-weight proanthocyanidins to which numerous beneficial health effects have been attributed [28]. Therefore, this study hypothesized that the addition of CFE as a functional ingredient to a meat matrix attenuates lipoprotein alteration induced by a high cholesterol/high saturated-fat diet in diabetic Wistar rats. In addition, we hypothesized that the CFE effects will be better when this functional ingredient is consumed a few weeks before STZ-NAD diabetic induction than they would be just after induction is confirmed. Therefore, the main objectives were to assess, in these rat models: (1) growth rate, (2) lipid and lipoprotein profiles, (3) VLDL oxidation, (4) plasma and liver arylesterase (AE) activities, (5) liver LDL receptor (*Ldlr*) by Western blot and immunohistochemistry analysis, and (6) to compare CFE effects before and after STZ-NAD diabetic induction.

## 2. Materials and Methods

### 2.1. Carob Fruit Extract (CFE)

CFE is a natural insoluble dietary fiber, which is obtained from carob pulp following the procedure described in patent WO2004/014150 [27] and Macho-González et al. [22]. Briefly, its major compound is total dietary fiber, 74–84%, of which the soluble fraction accounts for 1–3% and the insoluble one for 71–81%. CFE has a polyphenols concentration of non-extractable condensed tannins of 34–40% and soluble extractable polyphenols of 0.5–1%, according to patent WO2006/000551 [29].

### 2.2. Restructured Meat and Diet Preparation

Diets were prepared from a purified diet formulation (reference U8959, version 180; Panlab S.L.). Briefly, mixed minced meat (50% pork:50% veal) and lard were purchased at a local store. Restructured meat (RM) was prepared following the protocol described by Schultz-Moreira et al. [30]. For RM with CFE (CFE-RM), CFE (4 g/kg restructured meat) was homogenized with lean mixed meat. The resulting RMs were freeze-dried and ground in a chilled meat cutter (Stephan Universal Machine UM5; Stephan, Shóne Gmbh and Co.) following a standard procedure to obtain a powder product (Supplementary Table S1) [30]. For each kilogram of diet, 30% of RM, 5% of cellulose powder and 65% of a purified diet formulation were mixed and subsequently sieved 3 times until a completely homogenous powder was obtained. Diets were designed to contain the following: Control: 50 % energy from fat (20.4 % saturated fat), 36 % energy from carbohydrate, and 14 % energy from protein. Two experimental semisynthetic diets were prepared: (a) Diet containing the control-RM and 1.4% cholesterol and 0.2% cholic acid (98% purity) (Chol diet); and (b) identical to the Chol diet but containing CFE-RM (CFE diet). Diets designed with CFE-RM contained 1.14% less dietary cellulose with the objective of keeping similar total amount of fiber in diets (Table 1).

### 2.3. Experimental Design

Experiments were performed in compliance with directive 86/609/EEC of 24 November, 1986 (amended by Directive 2003/65/EEC of 22 July, 2003), on the protection of scientific research animals. The study was approved by the Spanish Science and Technology Advisory Committee (project AGL2014-53207-C2-2-R) and by the Ethics Committee of the Universidad Complutense de Madrid (Spain).

Twenty-four male Wistar rats aged two-months were obtained from Harlan S.L. (Barcelona, Spain) and housed in couples under a controlled temperature (22.3 ± 1.9 °C) and light (12-h light/dark cycle) at the Centro de Experimentación Animal of the University of Alcalá, Madrid, Spain (register no. ES280050001165). Food and tap water were provided ad libitum. Rats were divided into 3 groups of 8 animals each. At the 3rd week of the study T2DM was induced by intraperitoneal injection of streptozotocin (STZ, 65 mg/kg body weight, b.w.) and nicotinamide (NAD, 225 mg/kg b.w.) (both from Sigma-Aldrich, Madrid, Spain) [25,26]. Briefly, animals were divided into 3 groups: (1) Rats fed the Chol diet (D); (2) rats fed the CFE diet from the beginning of the study (ED); (3) rats fed the CFE diet when the diabetic state was confirmed (DE). The study lasted 8 weeks. 

In order to avoid inter-assay variations, overnight fasted rats were taken one at a time from each of the three groups at the end of experiment, anesthetized with isofluorane (5% *v*/*v*) and euthanized by extracting blood from the descending aorta with a heparinized syringe to cold tubes and placed in ice until processing.

### 2.4. Growth Rate and Fecal Fat Extraction

Food consumption was measured daily and body-weight once per week. The growth or conversion curves were individually made relating diet consumption (g) to body-weight gain (g). Apparent dietary digestibility was calculated according to the following formula: (feed intake (g)–fecal weight (g))/feed intake (g). Fecal fat was extracted and weighed according to Macho-González et al. [22].

### 2.5. Glycemia

Blood samples were obtained under fasting conditions in the middle of the 3rd week to confirm the diabetic state, and at the end of the eighth week of the experiment. Blood samples were placed in heparinized tubes and subsequently centrifuged to obtain plasma at 986× *g* for 10 min. Immediately, glycemia was quantified using a plate reader (SPECTROstar Nano, BMG LABTECH, Offenburg, Germany) at 492 nm, using the GOD kit (Spinreact, Barcelona, Spain) [21]. 

### 2.6. Lipoprotein Isolation

Plasma was separated from the whole blood by centrifugation for 20 min at 615× *g* and stored at 4 °C until lipoprotein isolation. The different lipoprotein fractions were obtained from 2 mL plasma by saline gradient ultracentrifugation (Beckman L8-70M) using an SW-40.1 rotor following a modification of the Terpstra et al. method [31], according to Olivero-David et al. [11]. Briefly, the tubes were centrifuged for 21 h 40 min at 272,000× *g* (40,000 rpm) at 4 °C. Isolation of the lipoprotein fractions was performed taking into account conventional density range for rats of the different lipoprotein classes (VLDL (ρ_20_ < 1.0063 g/mL), IDL (1.0063 < ρ_20_ < 1.019), LDL (1.019 < ρ_20_ < 1.057), and HDL (1.057 < ρ_20_ <1.21 g/mL)) [31].

### 2.7. Plasma Lipid Analysis and Lipoprotein Composition

Triglycerides, total cholesterol and phospholipids were quantified in plasma and lipoprotein fractions (VLDL, IDL, LDL, and HDL). The measurements were made in plate readers at 492 nm (SPECTROstar Nano, BMG LABTECH, Offenburg, Germany), using the Triglycerides-LQ, Cholesterol-LQ and Phospholipids kits (Spinreact, Barcelona, Spain) according to the manufacturer’s instructions. Total lipids were calculated as the sum of triglycerides, cholesterol and phospholipids [9,13]. The protein content of isolated lipoproteins was determined by the Bradford method [32]. The total mass of each lipoprotein fraction was calculated as the sum of total lipids plus proteins (both in mg/dL). The atherogenic index (AI) was determined as follows: (total cholesterol–HDL cholesterol)/HDL cholesterol [9,13].

### 2.8. Arylesterase Activity Measurement

Rat plasma AE activity was measured using simulated body fluid (SBF) as a buffer according to Nus et al. [33]. One unit of AE was defined as the phenol (mmol) formed from phenylacetate per minute. Reaction rates were monitored at 270 nm in thermostated quartz cuvettes with a 10 mm light path, using a spectrophotometer (SPECTROstar Nano). Liver AE was measured in liver extracts according to Garcimartín et al. [9] and results expressed in AE units/mg protein. Liver protein was measured according to Bradford method [32]. A blank of each sample without plasma or liver extract was made to correct the spontaneous hydrolysis of phenylacetate in SBF. Each measurement was performed in duplicate.

### 2.9. VLDL and Liver Oxidation (TBARS Assay)

VLDL and liver oxidation (VLDL-ox and liver-ox, respectively) were quantified as malondialdehyde while thiobarbituric acid reactive substances (TBARS) content was measured in accordance to the Mihara and Uchiyama method [34] as indicated by Garcimartín et al. [9].

### 2.10. LDL-Receptor Levels by Western Blotting and Immunohistochemistry

LDL-receptor (*Ldlr*) levels were analyzed in liver by western blot. Samples were homogenized with lysis buffer containing 10 mM Tris-HCl (pH 7.4), 1% SDS, 1 mM sodium vanadate and 0.01% protease inhibitor cocktail (all from Sigma-Aldrich). Liver protein was measured using the Lowry method [35]. Equal amounts of protein (30 μg) were separated at 150 V in 8% (*v*/*v*) polyacrylamide gel (SDS–PAGE) and, after migration, transferred to polyvinylidene fluoride (PVDF) membrane (GE Healthcare, Madrid, Spain) with Trans-Blot Turbo (Bio-Rad, Madrid, Spain) for 20 min and 2.5 mA. The membranes were blocked with 5% bovine serum albumin (BSA) for 1 h, and incubated overnight at 4 °C with primary antibodies anti-*Ldlr* (1:750) (sc-18823, Santa Cruz Biotechnology, Quimigen, Madrid, Spain) and anti-β-actin (1:30,000) (Sigma-Aldrich). Hybridization of antibodies was revealed by incubating the membranes with the appropriate secondary antibodies conjugated with peroxidase for 1h at room temperature. The chemiluminescence signal was detected using the ECL kit Select-kit (GE Healthcare, Madrid, Spain) and read in an ImageQuant LAS 500 (GE Healthcare, Madrid, Spain). Band-density was quantified using the Quantity One v.4.2.6 program and quantification was calculated referring to β-actin used as the loading control.

Paraffin-embedded liver sections were deparaffinized and rehydrated in a graded ethanol series. After retrieving citrate antigen and quenching endogenous peroxidase, sections were incubated with different primary antibodies overnight at 4 °C to determine the presence of *Ldlr* (Santa Cruz Biotechnology). The color reaction was developed with a polymerized horseradish peroxidase-conjugated secondary antibody and counterstained with hematoxylin. Fiji ImageJ processing software (U.S. National Institutes of Health, Bethesda, MD, USA) was used to count the stained vs. unstained pixels in each section. The immunolocalization of these proteins was studied by triplicate in eight different representative liver sections.

### 2.11. Statistical Analyses

Statistical analysis was performed using SPSS version 25.0 (SPSS Inc., Chicago, IL, USA). Results were expressed as mean ± SD. The effect of each independent variable was analyzed by means of an analysis of the variance (ANOVA) followed by T2-Tamhane or Bonferroni post-hoc test after assuming inequality or equality of variances, respectively. Differences in growth rate induced by diet were assessed by the ANCOVA test. The Chi-squared test was used to compare hypercholesterolemic or diabetic rat-distributions among groups. Differences were considered significant at *p* < 0.05.

## 3. Results

### 3.1. Growth Rate, Feed Consumption and Fecal Excretion

**Figure 1:**
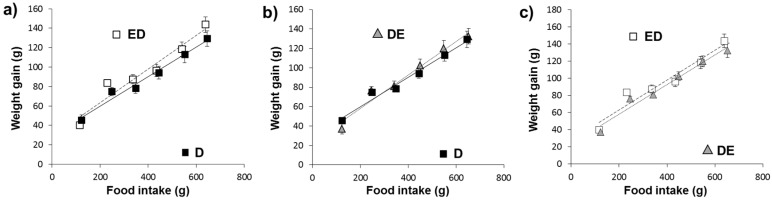
Growth rates of animals fed different diets. ED: Rats fed the Carob fruit extract (CFE)-diet since the beginning of the study; D: Rats fed the Chol-diet; DE: Rats fed the CFE-diet when the diabetic state was confirmed. For more abbreviations see footnotes of Table 1. Y = (slope with their SEM) * X + (intercept with their SEM), where Y is the body-weight gain and X is the food consumption. (**a**) ED [Y = (0.1789 ± 0.011) * X + (25.99 ± 1.66)] vs. D [Y = (0.149±0.004) * X + (26.98±2.10)] ANCOVA test *p* < 0.05; (**b**) DE [Y = (0.177 ± 0.005) * X + (23.76±1.92)] vs. D [Y = (0.149±0.004) * X + (26.98±2.10)] ANCOVA test *p* < 0.05; (**c**) ED [Y = (0.1789 ± 0.011) * X + (25.99 ± 1.66)] vs. DE [Y = (0.177 ± 0.005) * X + (23.76 ± 1.92)] ANCOVA test *p* > 0.05. R^2^ adjust for ED, D and DE were 0.94, 0.95 and 0.95, respectively.

Figure 1a–c shows the relationship between food intake and body-weight gain and the intercepts, slopes and significances found for the different groups. Diet significantly affected the growth curve. A significantly lower growth rate in D animals compared to ED and DE (ANCOVA, *p* < 0.05) was found

Table 2 summarizes the feed intake, growth rate, final weight and fecal excretions in the different rat groups. Fecal excretion, fecal moisture, fecal fat and dietary digestibility were significantly modified by diet (ANOVA at least, *p* = 0.004). Cholesterol intake was similar in all groups. Fecal excretion and moisture were significantly higher in ED and DE groups in comparison with D (*p* < 0.001). ED and DE vs. D rats showed a greater fecal fat amount but a lower food digestibility (*p* < 0.001). Non-significant differences (*p* > 0.05) were observed between ED group vs. DE group.

### 3.2. Plasma Glucose and Lipid Concentrations

Table 3 shows glycemia, plasma lipid concentrations, the AI and the cholesterol-to-phospholipid and cholesterol-to-HDL cholesterol ratios in the different groups. Except for glycemia at 3rd week, plasma phospholipids, and the cholesterol/phospholipid ratio; all other markers were significantly affected by diet (ANOVA at least *p* = 0.013).

Hyperglycemia (≥ 7 mmol/L) was found in 87.5% of ED and D rats, and 100% in DE rats at the 3rd week (chi square test *p* < 0.001). Marked hyperglycemia (>11.1 mmol/L) was found in all rats at the 8th week; however, ED and DE rats revealed significant lower glycemia than D rats (*p* < 0.001). DE rats showed significantly less final glycemia than ED rats (*p* < 0.05). D rats exhibited higher cholesterol than ED and DE (*p* < 0.001) and higher triglycerides than ED (*p* = 0.013). Hypercholesterolemia (total cholesterol ≥ 2.59 mmol/L) was found in 87.5% of D rats, 25% of DE rats but 0% of ED animals (chi square test *p* < 0.001). A higher cholesterol, AI, and cholesterol/HDL cholesterol ratio was found in DE rats vs. ED rats (*p* < 0.05).

### 3.3. Lipoprotein Composition

The composition of the different lipoprotein fractions in absolute values is shown in Table 4. Except for HDL-cholesterol and VLDL-total lipids most lipid components and total mass of VLDL, IDL, LDL and HDL fractions were significantly affected by diet (ANOVA *p* < 0.001). IDL proteins were affected by diet (*p* = 0.005). 

ED rats show a significantly (at least *p* < 0.05) lower content of cholesterol, phospholipids and total mass in all lipoprotein fractions except HDL and higher triglycerides in VLDL, lower total lipids in IDL and LDL, and lower protein in IDL than their D counterparts. DE rats showed significantly (at least *p* < 0.05) lower cholesterol in VLDL, lower cholesterol and phospholipids but higher triglycerides in IDL, lower cholesterol, phospholipids, total lipids and total mass in LDL than their D counterparts. 

VLDL fraction showed more cholesterol and phospholipids but fewer triglycerides in DE vs. ED rats. IDL fraction exhibited more triglycerides, phospholipids, total lipids, proteins and total mass in DE vs. ED rats. LDL fraction presented higher cholesterol, total lipids, and total mass in DE vs. ED rats. HDL fraction showed fewer phospholipids, total lipids and total mass in DE vs. ED rats.

### 3.4. Percentage Contribution of Lipids to Lipoprotein Composition

**Figure 2:**
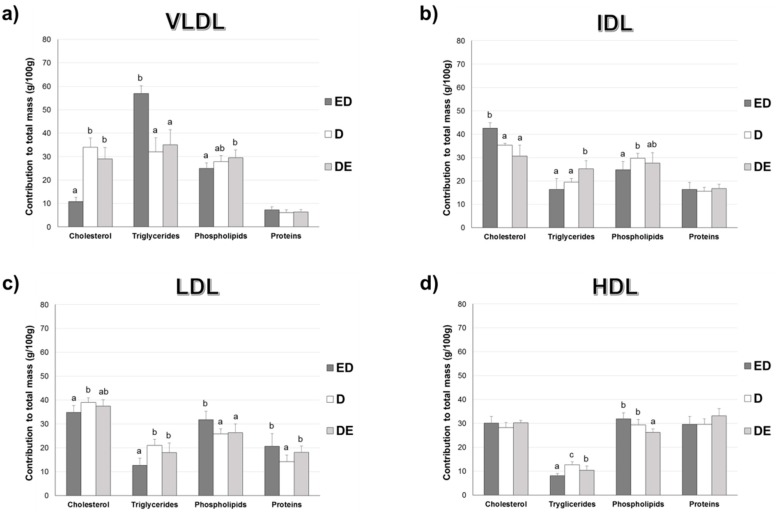
Percentage contribution (%) of proteins and the different lipids to the total mass of plasma VLDL, IDL, LDL and HDL fractions. Results are means ± SD (*n* = 8). ED: rats fed the CFE-diet since the beginning of the study; D: rats fed the Chol-diet; DE: rats fed the CFE-diet when the diabetic state was confirmed. For more abbreviations see footnotes of Table 1. (**a**) VLDL fraction composition: cholesterol (*p* < 0.001), triglycerides (*p* < 0.001), phospholipids (*p* = 0.010) and proteins (*p* > 0.05); (**b**) IDL fraction composition: cholesterol (*p* < 0.001), triglycerides (*p* = 0.009), phospholipids (*p* = 0.031) and proteins (*p* > 0.05); (**c**) LDL fraction composition: cholesterol (*p* = 0.009), triglycerides (*p* < 0.001), phospholipids (*p* = 0.013) and proteins (*p* < 0.08). (**d**) HDL fraction composition: cholesterol (*p* > 0.05), triglycerides (*p* < 0.001), phospholipids (*p* < 0.001) and proteins (*p* > 0.05). Labeled means for a variable without a common letter differ (*p* < 0.05; a < b < c; ab = a or b; ANOVA followed by the *post hoc* Bonferroni or T2 Tamhane test).

Figure 2 shows the percentage contribution of lipids and proteins to the total mass of plasma VLDL, IDL, LDL, and HDL fractions. Diet significantly affected all compositions of VLDL and IDL except proteins in both lipoproteins (ANOVA at least, *p* = 0.031); all LDL components (ANOVA at least, *p* < 0.013), and HDL triglycerides and phospholipids (*p* < 0.001).

ED rats had less cholesterol but more triglycerides (in percentage) in VLDL fraction than D rats (*p* < 0.001). Cholesterol contributed more while phospholipids less to the IDL total mass in ED rats vs. D rats. Cholesterol and triglycerides contributed less but phospholipids and proteins more to the total mass of LDL in ED vs. D rats. Triglycerides contributed less to the HDL fraction in ED vs. D rats.

DE rats show similar VLDL component contributions to total mass than D rats. Total triglycerides contributed more to total mass of IDL in DE vs. D rats. DE rats exhibited more proteins to LDL fraction than D groups. DE rats showed lower triglycerides and phospholipids contribution to the total HDL mass than D rats.

Cholesterol and phospholipids contributed more and triglyceride less to VLDL total mass in the DE vs ED rats. Cholesterol contributed less but triglycerides more to IDL particles in the DE vs. ED rats. Triglycerides and phospholipids contributed more to the LDL particles in the DE vs. ED rats. Triglycerides contributed more but phospholipids less to the HDL total mass in the DE vs. ED rats.

### 3.5. Arylesterase Activity, VLDL and Liver Oxidation

Plasma AE activity, the AE activity-to-plasma cholesterol ratio, liver AE activity, VLDL-ox and liver-ox are shown in Table 5. Diet significantly affected (ANOVA at least, *p* = 0.024) all parameters. Except for VLDL-ox, D rats showed lower plasma and liver AE, plasma AE/cholesterol ratio and higher liver-ox compared to ED and DE ones (*p* < 0.05). ED and DE showed higher plasma AE activity, plasma AE/cholesterol ratio, liver AE activity but lower liver oxidation respect to D (*p* < 0.001). Likewise, a significantly higher plasma AE activity and in the plasma AE/cholesterol ratio (*p* < 0.001) and lower VLDL-ox (*p* < 0.05) were observed in DE compared to D animals.

### 3.6. Liver Macroscopic Aspect

Liver hypertrophy and steatosis was clearly observed. However, ED rats followed by DE animals display a considerably steatosis reduction in macroscopic observation (Supplementary Figure S1).

### 3.7. Liver LDL Receptor Levels

**Figure 3:**
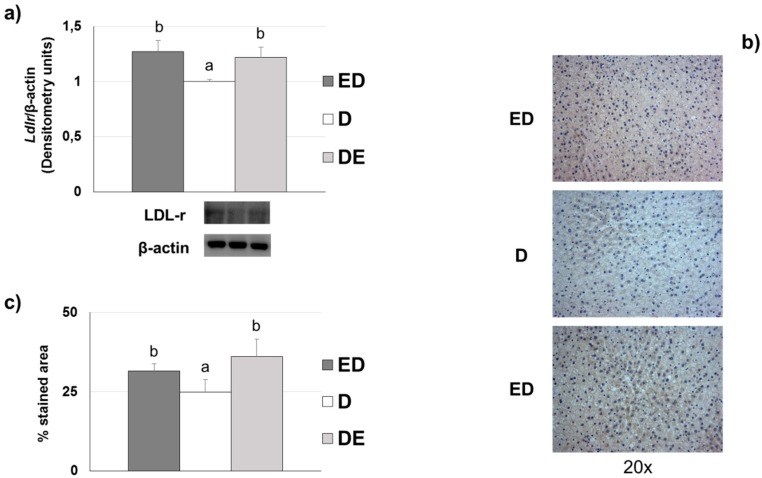
*Ldlr* levels liver localization in the different diabetic groups fed the three experimental diets (*n* = 8 rats/group). ED: rats fed the CFE-diet since the beginning of the study; D: rats fed the Chol-diet; DE: rats fed the CFE-diet when the diabetic state was confirmed. For more abbreviations see footnotes of Table 1. (**a**) Representative blots from liver *Ldlr* levels. Analysis of the variance (ANOVA), *p* < 0.001. Labeled means for a variable without a common letter differ (*p* < 0.05; a < b; ANOVA followed by the *post hoc* T2 Tamhane test). (**b**) The immunolocalization of *Ldlr* was studied by triplicate in eight different representative liver sections (20×). (**c**) Percentage stained area of hepatocytes *Ldlr*. Analysis of the variance (ANOVA), *p* < 0.001. Labeled means for a variable without a common letter differ (*p* < 0.05; a < b; ANOVA followed by the *post hoc* Bonferroni test).

Diet significantly affected (ANOVA, *p* < 0.001) the hepatic *Ldlr* levels. ED and DE rats showed higher levels by Western blot of the *Ldlr* compared to D rats (Figure 3a). Non-significant differences (*p* > 0.05) were observed between ED group vs. DE group. Figure 3b,c show the immunohistochemistry data of *Ldlr* in liver sections. All groups displayed different *Ldlr* immunohistochemical staining levels (ANOVA, *p* < 0.001). ED and DE rats exhibited significantly higher *Ldlr* levels than D counterparts (*p* < 0.05).

## 4. Discussion

The results described show the effect of CFE included in a meat matrix as a nutritional strategy to reduce the qualitative and quantitative lipoprotein alterations in T2DM rats fed a high saturated-fat/high-cholesterol diet. CFE prevented the negative effects of this atherogenic diet by reducing basal glycemia, cholesterolemia, triglyceridemia and liver oxidation, at the same time as improving the lipoprotein profile [11,36] and increasing the AE activity, fecal fat excretion and *Ldlr* levels in STZ-NAD diabetic rats. These results are relevant given the concern between high meat-consumption and degenerative diseases prevalence [1], although it has been observed that meat-product consumption can improve GLP-1 and GPI, incretins that are affected in T2DM subjects [4,6,7]. Therefore, the possibility of reducing the negative effects high consumption of meat products in the frame of an atherogenic diet by including the CFE as a functional ingredient in the meat product can be suggested. 

**Figure 4:**
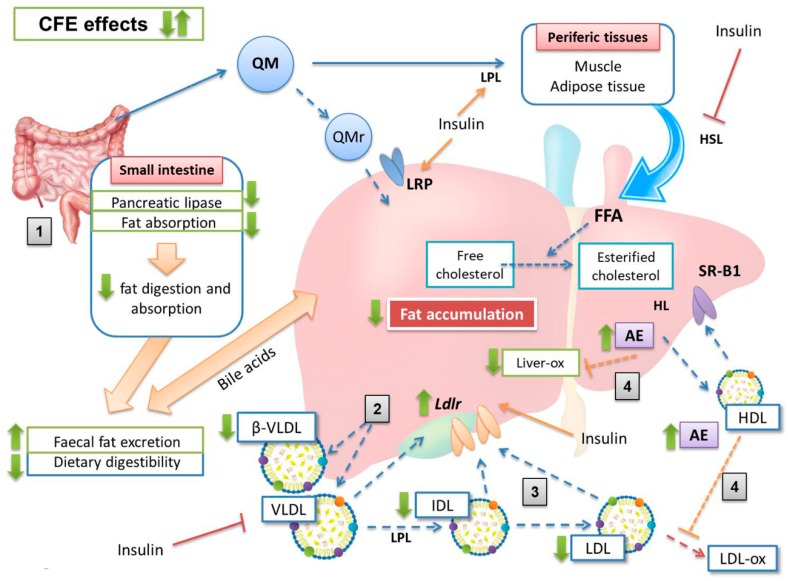
Proposed mechanism of the carob fruit extract (CFE) effects on lipoprotein metabolism. The diabetogenic status induced by STZ-NAD administration in addition to the atherogenic diet give raise to a hyperglycemic status compatible with a decrease in plasma insulin levels [25]. The insulinemia reduction implies higher FFA release increased VLDL production and *Ldlr* decrease [6]. The cholesterol-enriched diet produces increased level of cholesterol-enriched VLDL (β-VLDL) [9,13]. Green arrow indicates the increase (↑) or decrease (↓) for each marker assigned. Stages: (1) CFE increases the fat excretion of the atherogenic diet, mainly by decreasing dietary digestibility (reduction of fat digestion and absorption); (2) the lower plasma cholesterol and triglycerides in CFE groups correspond to a lower production of triglyceride enriched-VLDL and decreased β-VLDL synthesis; (3) CFE decreases the IDL and LDL levels, as a consequence of a higher hepatic *Ldlr* levels; (4) CFE increases the plasma and liver AE activities, reducing the VLDL and liver oxidation observed in the non-CFE rats. AE, arylesterase; CFE, carob fruit extract; FFA, free fatty acids; HDL, high-density lipoproteins; HL, hepatic lipase; HSL, hormone-sensitive lipase; IDL, intermediate-density lipoproteins; LDL, low-density lipoproteins; *Ldlr*, low density lipoprotein receptor; LRP, low density lipoprotein receptor-related protein; QM, chylomicrons; QMr, remnant chylomicrons; SR-B1, hepatic scavenger receptor B-I; VLDL, very low-density lipoproteins; β-VLDL, cholesterol-enriched very low-density lipoproteins.

Based on the evidence of the present and previous related studies, a mechanism explaining results for CFE-RM consumption in STZ-NAD diabetic rats is proposed (Figure 4).

All study diets were well accepted, as observed in the intake and growth data, which are consistent with results of our group studying different RM on cholesterol fed-rats [10,11,12]. Although the final weights of the study animals were very similar among all groups, a lower growth slope in D rats was found, which is typical of animals fed cholesterol-enriched diets [9,13,30,37]. This growth slope was higher in the ED and DE groups. On the other hand, CFE diet induced greater fecal excretion and higher fecal fat content with respect to rats fed the control-RM diet, giving rise to dietary digestibility reduction. This effect is justified because CFE-composition is based on insoluble fiber and proanthocyanidins, which leads to a reduction in fat and carbohydrate digestions, as observed by Macho-González et al. in postprandial studies developed in healthy rats fed the same extract [21,22]. Despite the higher fecal excretions of ED and DE rats with respect to D animals, the final body weight tended (non-significantly; *p* > 0.05) to increase, suggesting that CFE-fed rat release less fatty acids from adipose tissue, improving the diabetogenic situation induced by STZ-NAD administration [6,7].

One of the most stressful findings of the present paper is the significant reduction in fasting glycemia observed in STZ-NAD animals fed with the CFE (13% in ED rats and 23% in DE rats) vs. the D counterparts. As discussed, CFE was able to reduce postprandial glycemia in chow-diet rats [21]. Some fiber compounds have been found to reduce glycemia and lipemia [38]. Cholesterol-enriched diets induced moderate hypercholesterolemia in group D (87.5% of D rats show cholesterol levels ≥ 2.59 mmol/L or ≥ 100 mg/dL, defined as cut-off point) but 0% in ED and only 25% of DE rats displayed this level, suggesting the hypocholesterolemic effect of the CFE amount assayed [36]. These animals also show a modified lipoprotein profile according to the AI and the cholesterol/phospholipids and cholesterol/HDL-cholesterol ratios that were in line with previous results obtained by our group [9,13] allowing us to agree with Ruiz-Roso et al. [39] who showed that carob fruit reduced plasma cholesterol in hypercholesterolemic subjects. Results on lipoprotein fractions indicate that D animals showed the typical lipoprotein profile of hypercholesterolemic rats with the presence of cholesterol-enriched and triglyceride-depleted VLDL (β-VLDL) [11,12,13,40,41]. In contrast, the ED group displayed more normalized, triglyceride-enriched VLDL. These β-VLDL have been defined as atherogenic lipoproteins for the rat [42]. In addition, the presence of increased IDL and LDL particles was also clearly observed. According to Sánchez-Muniz et al., as each LDL particle contains only one ApoB100 molecule, it can be calculated that ED presented 19% and DE 5% less average LDL particles than D counterparts [43]. The LDL levels in D rats also correspond to the hypercholesterolemic diet effect itself, as observed in previous studies by our group [9,10,11]. These LDL values also seem related to the higher triglyceride-availability in D rats, as well as a clear decrease in catabolism, as a consequence of a lower expression of the hepatic *Ldlr*, which implies a clear reduction of CVD risk by CFE-RM-consumption mainly in the ED group. In situations where *Ldlr* have downregulated, LDL-removal decreases and VLDL remnant-removal should also decrease. Conversion of VLDL to LDL would then increase. Both of these circumstances would also increase LDL concentrations [44]. The triglyceride contribution to HDL total mass was higher in D rats vs. ED and DE, partially due to the higher levels of plasma triglycerides observed, that in turn favors the production of VLDL and promotes triglyceride enrichment of HDL particles [6]. The *Ldlr* reduction may also be a consequence of the greater synthesis of LDL associated with the hypercholesterolemic diets. The lesser liver *Ldlr* amount in D animals seems linked to a mechanism to avoid cholesterol-accumulation in the liver [9,13] but is also probably related to an insulin-resistance status in these rats, a fact observed in STZ-NAD animals fed a high-saturated diet [45]. Finally, D animals show higher VLDL-ox and liver-ox amounts while lower plasma and liver AE, in line with the reduced antioxidant defense observed in diabetic animals and patients [46].

As described, these alterations in the lipoprotein composition were partially reversed in the CFE-RM animals, mainly in ED rats that show lower cholesterol and more triglycerides contributing to the VLDL total mass [10]. Likewise, it can be suggested that hepatic LDL and LDL re-uptake was more effectively performed in CFE-RM groups, basically due to the higher levels of *Ldlr* found. These results may be related to an increase in cholesterol esterification as a strategy to increase *Ldlr* levels [47], as well as to the possible higher plasma insulin values associated with proanthocyanidins-consumption [48]. However, CFE was not able to increase HDL levels. It has been proposed that in hypercholesterolemic rats the decrease in HDL levels is related to the uptake of these lipoproteins by hepatic scavenger receptor B-I (SR-BI) in order to increase biliary excretion and reduce plasma cholesterol levels [49].

The data of the present study suggest a greater VLDL oxidation and greater liver lipid peroxidation susceptibilities in D group, both associated with the consumption of hypercholesterolemic diet and the diabetic induction [9,50,51]. However, animals consuming CFE-RM showed reduction in VLDL and liver oxidations, as a consequence of the marked antioxidant properties of proanthocyanidins [52]. These results also seem be associated with the higher plasma and liver AE activities of these groups, suggesting that proanthocyanidin active metabolites could increase the antioxidant defense. In the same way, as serum paraoxonase 1 (PON1) is synthesized in the liver, the biggest changes associated with CFE-consumption was observed in this organ. Estrada-Luna et al. observed PON1 expression and activity increases in mice fed pomegranate juice in the frame of a high-fat diet [53]. Rock et al. also observed a PON1 activity increase in diabetic patients who ingested a pomegranate-rich tannin preparation [54]. Therefore, all these data taken together would justify a lower susceptibility to oxidation and a lower atherosclerosis risk in CFE groups [55,56].

Despite the positive results observed, this study presents some possible limitations: (1) only one dose of CFE was tested, (2) only one dose of STZ was employed, (3) the study lasted only eight weeks, (4) the study was performed only on growing male Wistar rats, and (5) rat lipoprotein profile was not assessed at the beginning of the study.

## 5. Conclusions

Results show for the first time that CFE-RM reduces the negative effects of an atherogenic diet, improving glycemia, lipemia, AE activity, *Ldlr* levels and lipoprotein profiles by reducing the presence of β-VLDL, IDL and LDL which leads to a reduction in CDV risk. Therefore, we can suggest that CFE is an adequate functional ingredient to be included in a meat matrix aimed to be preferably consumed by prediabetes and T2DM patients allowing them to get the high nutrient content of these food products and to correct lipoprotein alterations.

## Figures and Tables

**Table 1:** Composition of the experimental diets fed to Wistar rats.

Dietary Components	Chol-Diet	CFE-Diet
Protein, %	17.03	17.03
Fat, %	27.02	27.02
Cholesterol, g/kg	9.83	9.83
SFA:MUFA:PUFA ratio	2.76/2.99/1	2.76/2.99/1
Energy content *, MJ/kg	20.36	20.36
**Ingredients, g/kg**		
Sucrose	68.25	68.25
Corn starch	275.73	275.73
Casein	94.25	94.25
Maltodextrin	94.25	94.25
Cellulose	48.86	48.86
PM 205B SAFE	50.05	50.05
PV 200 SAFE	7.15	7.15
Soybean oil	47.91	47.91
L-Cysteine	2.02	2.02
Cholesterol	9.1	9.1
Cholic acid	1.3	1.3
Freeze-dried restructured meat	301.14	301.14

* Data were calculated according to the energy equivalents for carbohydrate (16.73 kJ/g (4.0 kcal/g)), fat (37.65 kJ/g (9.0 kcal/g)), and protein (16.73 kJ/g (4.0 kcal/g)). Chol-diet, diet containing the control-RM and 1.4% cholesterol and 0.2% cholic acid; CFE-diet, diet containing the carob fruit extract-RM (CFE-RM) and 1.4% cholesterol and 0.2% cholic acid. SFA, Saturated fatty acids; MUFA, Monounsaturated fatty acids; PUFA, Polyunsaturated fatty acids; PM 205B SAFE, Mineral mix; PV 200 SAFE, Vitamin Mix.

**Table 2:** Feed intake, anthropometric characteristics and fecal excretion of animals fed the experimental diets.

				ANOVA
	ED	D	DE	*p*
**Cholesterol intake (g/wk)**	1.07 ± 0.04	1.08 ± 0.08	1.09 ± 0.08	*p* > 0.05
Growth rate *	0.18 ± 0.03 ^b^	0.15 ± 0.01 ^a^	0.18 ± 0.01 ^b^	*p* = 0.019
**Final weight (g)**	380.1 ± 39.3	366.6 ± 32.3	380.4 ± 22.3	*p* > 0.05
Fecal excretion (g/wk) **	14.10 ± 1.43 ^b^	11.16 ± 0.43 ^a^	13.57 ± 1.69 ^b^	*p* = 0.001
**Fecal moisture (%)**	18.56 ± 1.55 ^b^	14.04 ± 1.66 ^a^	16.62 ± 1.40 ^b^	*p* < 0.001
Fecal fat (mg/g feces) **	226.6 ± 26.93 ^b^	170.6 ± 10.07 ^a^	236.5 ± 25.20 ^b^	*p* < 0.001
Dietary digestibility ***	0.85 ± 0.01 ^a^	0.88 ± 0.01 ^b^	0.86 ± 0.02 ^ab^	*p* = 0.004

Results are means ± SD (*n* = 8). Labeled means in a row without a common letter differ (*p* < 0.05; a < b < c; ab = a or b; ANOVA followed by the Bonferroni or T2 Tamhane *post hoc* test). ED: rats fed the CFE-diet since the beginning of the study; D: rats fed the Chol-diet; DE: rats fed the CFE-diet when the diabetic state was confirmed. For more abbreviations see footnotes of Table 1. * Growth rate: Conversion rate relating food consumption (g) to body-weight gain (g). ** Data are dry matter weights; *** Dietary digestibility = (feed intake−feces)/feed intake.

**Table 3:** Plasma lipids, cholesterol: phospholipid and cholesterol: HDL-cholesterol ratio and atherogenic index of animals fed the experimental diets.

				ANOVA
	ED	D	DE	*p*
**Glycemia 3rd week (mmol/L)**	8.02 ± 0.57	8.04 ± 0.96	8.27 ± 0.70	*p* > 0.05
**Glycemia 8th week (mmol/L)**	15.76 ± 0.76 ^b^	18.11 ± 1.65 ^c^	13.94 ± 1.13 ^a^	*p* < 0.001
**Total cholesterol (mmol/L)**	2.26 ± 0.12 ^a^	2.95 ± 0.22 ^c^	2.57 ± 0.16 ^b^	*p* < 0.001
**Triglycerides (mmol/L)**	0.74 ± 0.05 ^a^	0.87 ± 0.11 ^b^	0.71 ± 0.13 ^ab^	*p* = 0.013
**Phospholipids (mmol/L)**	1.14 ± 0.13	1.28 ± 0.09	1.26 ± 0.17	*p* > 0.05
Total lipids (mg/dL) *	235 ± 14.1 ^a^	282 ± 29.8 ^b^	252 ± 24.6 ^ab^	*p* = 0.003
**Cholesterol:phospholipids (mol/mol)**	1.01 ± 0.09	1.13 ± 0.10	1.02 ± 0.16	*p* > 0.05
**Cholesterol:HDL cholesterol (mol/mol)**	1.65 ± 0.29 ^a^	2.37 ± 0.45 ^b^	2.10 ± 0.18 ^b^	*p* < 0.001
Atherogenic index **	0.65 ± 0.29 ^a^	1.37 ± 0.45 ^b^	1.10 ± 0.18 ^b^	*p* < 0.001

Results are means ± SD (*n* = 8). Labeled means in a row without a common letter differ (*p* < 0.05; a < b < c; ab = a or b; ANOVA followed by the Bonferroni or T2 Tamhane *post hoc* test). ED: rats fed the CFE-diet since the beginning of the study; D: rats fed the Chol-diet; DE: rats fed the CFE-diet when the diabetic state was confirmed. For more abbreviations see footnotes of Table 1. * Total lipids: cholesterol + triglycerides + phospholipids; ** Atherogenic index = (total cholesterol–HDL cholesterol)/HDL cholesterol. To transform mmol/L into mg/dL of cholesterol, triglycerides, and phospholipids, multiply data by 38.68, 89.0, and 75.0, respectively.

**Table 4:** Lipoprotein component concentrations in plasma of animals fed the experimental diets.

				ANOVA
Lipoprotein	ED	D	DE	*p*
**Cholesterol, mmol/L**				
**VLDL**	0.22 ± 0.03 ^a^	0.78 ± 0.09 ^c^	0.62 ± 0.12 ^b^	*p* < 0.001
**IDL**	0.44 ± 0.08 ^a^	0.55 ± 0.03 ^b^	0.41 ± 0.05 ^a^	*p* < 0.001
**LDL**	0.16 ± 0.03 ^a^	0.34 ± 0.06 ^c^	0.24 ± 0.01 ^b^	*p* < 0.001
**HDL**	1.40 ± 0.16	1.28 ± 0.19	1.22 ± 0.08	*p* > 0.05
**Triglycerides, mmol/L**				
**VLDL**	0.48 ± 0.07 ^b^	0.30 ± 0.07 ^a^	0.32 ± 0.08 ^a^	*p* < 0.001
**IDL**	0.07 ± 0.02 ^a^	0.13 ± 0.02 ^b^	0.14 ± 0.05 ^b^	*p* < 0.001
**LDL**	0.02 ± 0.01 ^a^	0.07 ± 0.02 ^c^	0.05 ± 0.01 ^b^	*p* < 0.001
**HDL**	0.15 ± 0.01 ^a^	0.23 ± 0.02 ^b^	0.17 ± 0.03 ^a^	*p* < 0.001
**Phospholipids, mmol/L**				
**VLDL**	0.25 ± 0.03 ^a^	0.31 ± 0.03 ^b^	0.31 ± 0.03 ^b^	*p* = 0.001
**IDL**	0.12 ± 0.03 ^a^	0.23 ± 0.01 ^c^	0.18 ± 0.04 ^b^	*p* < 0.001
**LDL**	0.07 ± 0.01 ^a^	0.11 ± 0.01 ^b^	0.08 ± 0.02 ^a^	*p* < 0.001
**HDL**	0.72 ± 0.02 ^c^	0.64 ± 0.06 ^b^	0.52 ± 0.04 ^a^	*p* < 0.001
Total lipids *, mg/dL				
**VLDL**	69.72 ± 7.67	79.50 ± 7.13	73.54 ± 8.67	*p* > 0.05
**IDL**	31.32 ± 4.69 ^a^	48.41 ± 2.57 ^c^	41.35 ± 4.28 ^b^	*p* < 0.001
**LDL**	13.57 ± 2.14 ^a^	27.70 ± 4.01 ^c^	19.24 ± 1.25 ^b^	*p* < 0.001
**HDL**	119.54 ± 5.82 ^b^	115.94 ± 11.30 ^b^	99.47 ± 7.11 ^a^	*p* < 0.001
**Proteins, mg/dL**				
**VLDL**	5.43 ± 0.83	5.15 ± 0.86	4.89 ± 0.52	*p* > 0.05
**IDL**	6.31 ± 2.20 ^a^	8.93 ± 1.32 ^b^	8.26 ± 0.96 ^ab^	*p* = 0.005
**LDL**	3.64 ± 1.34	4.51 ± 0.93	4.27 ± 0.76	*p* > 0.05
**HDL**	50.8 ± 8.94	48.8 ± 5.80	49.3 ± 5.79	*p* > 0.05
Total mass **, mg/dL				
**VLDL**	75.15 ± 6.33 ^a^	84.65 ± 6.36 ^b^	78.43 ± 8.62 ^ab^	*p* = 0.045
**IDL**	37.63 ± 6.48 ^a^	57.34 ± 3.13 ^c^	49.61 ± 4.73 ^b^	*p* < 0.001
**LDL**	17.21 ± 2.92 ^a^	32.22 ± 4.32 ^c^	23.51 ± 1.36 ^b^	*p* < 0.001
**HDL**	170.3 ± 12.19 ^b^	164.7 ± 14.92 ^ab^	148.8 ± 7.54 ^a^	*p* < 0.001

Results are means ± SD (*n* = 8). Labeled means in a row without a common letter differ (*p* < 0.05; a < b < c; ab = a or b; ANOVA followed by the Bonferroni or T2 Tamhane *post hoc* test). ED: rats fed the CFE-diet since the beginning of the study; D: rats fed the Chol-diet; DE: rats fed the CFE-diet when the diabetic state was confirmed. VLDL, very low-density lipoproteins; IDL, intermediate-density lipoproteins; LDL, low-density lipoproteins; HDL, high-density lipoproteins. For more abbreviations see footnotes of Table 1. * Total lipids: cholesterol + triglycerides + phospholipids; ** Total mass: total lipids + proteins.

**Table 5:** Plasma and liver arylesterase (AE) activity and VLDL and liver oxidation of animals fed the experimental diets.

				ANOVA
	ED	D	DE	*p*
Plasma AE (U/L) *	237 ± 43.06 ^b^	163 ± 29.56 ^a^	368 ± 58.59 ^c^	*p* < 0.001
Plasma AE:cholesterol (U/mg) **	0.29 ± 0.05 ^b^	0.15 ± 0.02 ^a^	0.39 ± 0.05 ^c^	*p* < 0.001
**Liver AE (U/g protein)**	7.70 ± 0.97 ^b^	5.36 ± 0.83 ^a^	10.93 ± 3.28 ^b^	*p* < 0.001
**VLDL-ox (TBARS, mg MDA/L)**	2.31 ± 0.29 ^ab^	2.58 ± 0.16 ^b^	2.22 ± 0.23 ^a^	*p* = 0.024
**Liver-ox (TBARS, mg MDA/mg protein)**	2.63 ± 0.58 ^a^	3.63 ± 0.25 ^b^	2.49 ± 0.33 ^a^	*p* < 0.001

Results are means ± SD (*n* = 8). Labeled means in a row without a common letter differ (*p* < 0.05; a < b < c; ab = a or b; ANOVA followed by the Bonferroni or T2 Tamhane *post hoc* test). ED: rats fed the CFE-diet since the beginning of the study; D: rats fed the Chol-diet; DE: rats fed the CFE-diet when the diabetic state was confirmed. For more abbreviations see footnotes of Table 1. * One unit of AE was defined as mmols of phenol formed from phenylacetate per minute; ** Units AE (U/L)/total cholesterol (mg/L).

## Supplementary Materials

The following are available online at http://www.mdpi.com/2072-6643/11/2/332/s1, Table S1: Composition of the Restructured Meat (RM) incorporated into the experimental diets fed to male Wistar rats, Figure S1: Macroscopic aspect of a representative liver of the experimental groups.

## Abbreviations

AE, arylesterase; AI, atherogenic index; CDV, cardiovascular disease; CFE, carob fruit extract; HDL, high-density lipoprotein; IDL, intermediate-density lipoproteins; LDL, low-density lipoprotein; *Ldlr*, LDL receptor; NAD, nicotinamide; PON1, paraoxonase 1; RM, restructured meat; STZ, streptozotocin; T2DM, Type 2 Diabetes Mellitus; VLDL, very low-density lipoprotein.
