# Supplementary material for: Can Carob-Fruit-Extract-Enriched Meat Improve the Lipoprotein Profile, VLDL-Oxidation, and LDL Receptor Levels Induced by an Atherogenic Diet in STZ-NAD-Diabetic Rats?
Source: Nutrients. 2019 Feb 3;11(2):332. doi: 10.3390/nu11020332 (PMC6413123; doi:10.3390/nu11020332)
Supplement: Supplementary file 1 [file nutrients-11-00332-s001.pdf]

Supplementary

Table S1. Composition of the Restructured Meat (RM) incorporated into the experimental diets fed to male Wistar rats.

Restructured meat components	Control-RM	CFE-RM
Protein, %	13.1	13.1
Fat, %	38.3	38.3
Water, %	46.9	46.9
Cholesterol, g/kg	0.74	0.74
SFA/MUFA/PUFA ratio	41.2/43.5/8.5	41.2/43.5/8.5
Ingredients		
Lean pork, g/kg	663.1	663.1
Lard, g/kg	331.1	331.1
Na, g/kg	0.5	0.5
STP, g/kg	0.1	0.1
Sodium nitrite, g/kg	1.2	1.2
CFE, g/kg	0.0	4
Cellulose	4	0.0

[1]Control Restructured Meat control (RM), Restructured Meat supplemented with CFE (CFE-RM). STP, sodium tripolyphosphate.

Figure S1.

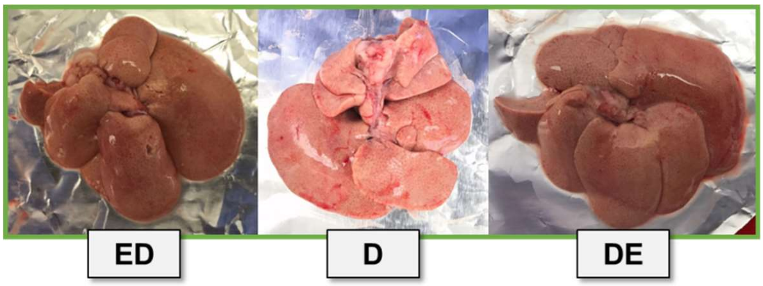

Figure S1. Macroscopic aspect of a representative liver of the experimental groups. Liver macroscopic aspect of the different diabetic groups fed the three experimental diets (n=8 rats/group). ED: rats fed the CFE-diet since the beginning of the study; D: rats fed the Chol-diet; DE: rats fed the CFE-diet when the diabetic state was confirmed.
